# Cross species extrapolation of the disruption of thyroid hormone synthesis by oxyfluorfen using in vitro data, physiologically based pharmacokinetic (PBPK), and thyroid hormone kinetics models

**DOI:** 10.1016/j.crtox.2023.100138

**Published:** 2023-11-23

**Authors:** Rhylee Decrane, Tammy Stoker, Ashley Murr, Jermaine Ford, Hisham El-Masri

**Affiliations:** a ORAU, Oak Ridge, TN, United States; b USEPA, ORD, CPHEA, RTP, NC, United States; c USEPA, ORD, CCTE, RTP, NC, United States

**Keywords:** Thyroid, PBPK, Biological modeling, Oxyfluorfen

## Abstract

The thyroid hormones play key roles in physiological processes such as regulation of the metabolic and cardiac systems as well as the development of the brain and surrounding sympathetic nervous system. Recent efforts to screen environmental chemicals for their ability to alter thyroid hormone synthesis, transport, metabolism and/or function have identified novel chemicals that target key processes in the thyroid pathway. One newly identified chemical, oxyfluorfen, is a diphenyl-ether herbicide used for control of annual broadleaf and grassy weeds in a variety of tree fruit, nut, vine, and field crops. Using in vitro high-throughput screening (HTS) assays, oxyfluorofen was identified to be a potent inhibitor of the thyroidal sodium-iodide symporter (NIS). To quantitatively assess this inhibition mechanism in vivo, we extrapolated in vitro NIS inhibition data to in vivo disruption of thyroid hormones synthesis in rats using physiologically based pharmacokinetic (PBPK) and thyroid hormone kinetics models. The overall computational model (chemical PBPK and THs kinetic sub-models) was calibrated against in vivo data for the levels of oxyfluorfen in thyroid tissue and serum and against serum levels of thyroid hormones triiodothyronine (T3) and thyroxine (T4) in rats. The rat thyroid model was then extrapolated to humans using human in vitro HTS data for NIS inhibition and the chemical specific hepatic clearance rate in humans. The overall species extrapolated PBPK-thyroid kinetics model can be used to predict dose–response (% drop in thyroid serum levels compared to homeostasis) relationships in humans. These relationships can be used to estimate points of departure for health risks related to a drop in serum levels of TH hormones based on HTS assays in vitro to in vivo extrapolation (IVIVE), toxicokinetics, and physiological principles.

## Highlights

• A computational model combining kinetics of the oxyfluorofen, and thyroid hormones synthesis, distribution, and clearance was developed for rats.
• The computational model was calibrated against serum levels of the thyroid hormones generated from the rat experimental studies.
• The computational model was extrapolated to humans to develop a dose–response relationship between oral doses of oxyfluorofen and precent drop of serum levels of thyroxine (T4)

## Graphical abstract

**Figure:**
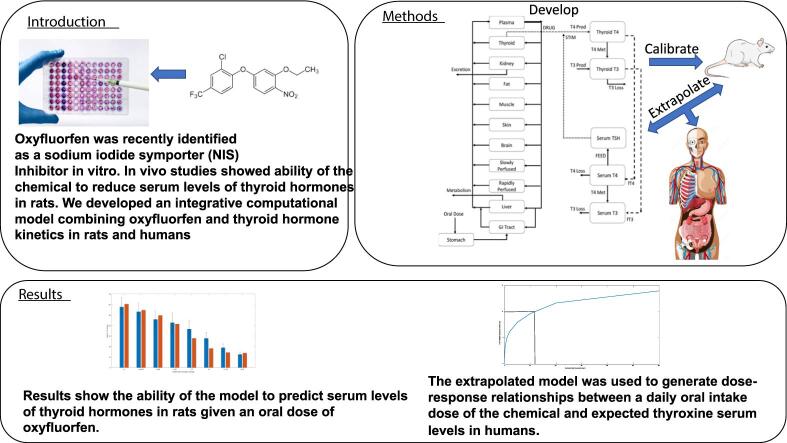

## Introduction

Triiodothyronine (T3), and thyroxine (T4) are the main thyroid hormones (THs) involved in many biological processes such as cellular growth development, and metabolism (Bassett et al., 2003, Yen, 2001). Circulating levels of THs in vivo are influenced by complex feedback mechanisms regulated by the hypothalamus-pituitary-thyroid (HPT) axis (Zoeller et al., 2007). These processes include the secretion of thyrotropin-releasing hormone (TRH) from the hypothalamus to the pituitary gland, which then leads to the release of thyroid-stimulating hormone (TSH) to stimulate the secretion of thyroid hormones from the thyroid. Synthesis of THs occurs between the colloid (a protein-rich region), and follicular cells (the functional units of the thyroid) (Zoeller et al., 2007). Critically, THs synthesis requires the iodination of two tyrosine molecules derived from the glycoprotein thyroglobulin. This process takes place when active iodide (I − ) is transported to thyroid tissue and is subsequently organified by the thyroid peroxidase (TPO) to form thyroid hormones and iodinated proteins (Ajjan et al., 1998, Spitzweg et al., 1998). Active iodide (I^-^) transport in both the thyroid and some extrathyroidal tissues is mediated by the Na+/I^-^ symporter (NIS). NIS resides in the basolateral membrane of thyroid epithelial cells and simultaneously transports two Na + and one I^-^ from extracellular fluid (plasma) into the thyroid epithelial cell (Spitzweg et al., 2000).

**Fig. 1:**
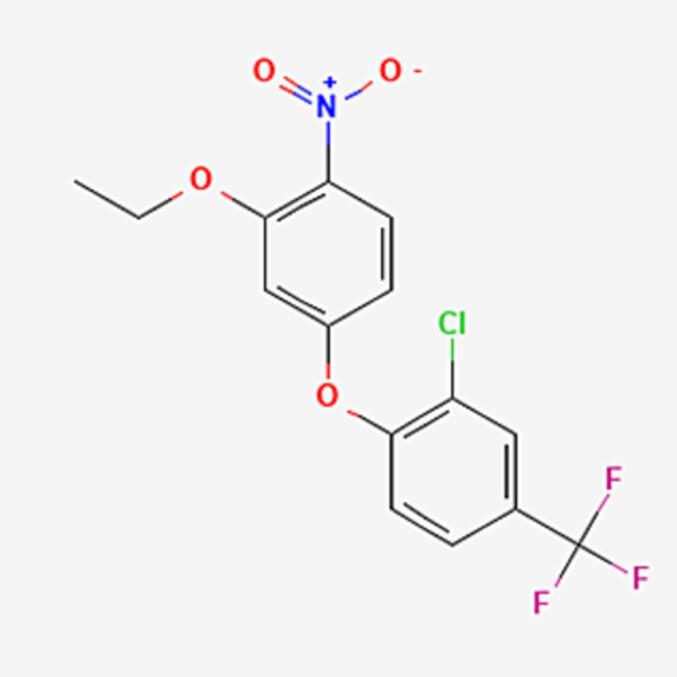
Chemical structure for Oxyfluorfen.

Disruption of THs serum levels in vivo has been associated with several adverse outcomes including abnormal brain development, learning impairment, and heart defects in humans and animal models (Bernal, 2017, Gilbert, 2011, Korevaar et al., 2016, Vale et al., 2019, Wu et al., 2006). Environmental chemicals have been shown to interfere with thyroid hormones regulation synthesis, and serum levels (Boas et al., 2009, Paul Friedman et al., 2016). For example, a listing of chemicals with their potential to inhibit THs synthesis in vitro via NIS inhibition is given in Buckalew et al. (2020). Using high throughput screening (HTS) assays, chemicals were tested in vitro using Fischer rat thyroid follicular cells (FRTL-5) applying the high-throughput radioactive iodide uptake (RAIU) assay based on an earlier chemical list generated for potential human NIS inhibitors using the human hNIS-HEK293T-EPA cell line (Buckalew et al., 2020). One newly identified chemical with potential to inhibit NIS in vitro, oxyfluorfen, is a diphenyl-ether herbicide used for control of annual broadleaf and grassy weeds in a variety of tree fruit, nut, vine, and field crops (Fig. 1). Oxyfluorfen was ranked within the top 20 of the 293 ToxCast phase I tested chemicals for inhibition of NIS in vitro when compared to the typical inhibitor sodium perchlorate (Wang et al., 2018). Humans may be exposed to oxyfluorfen in food and drinking water since oxyfluorfen may be applied directly to growing crops and application may result in oxyfluorfen reaching surface and ground sources of drinking water (https://www3.epa.gov/pesticides/chem_search/reg_actions/reregistration/fs_PC-111601_1-Oct-02.pdf).

Translation of the in vitro potential for oxyfluorfen to inhibit I^-^ uptake into the thyroid tissue, and the disruption of THs synthesis in vivo requires the inhibitory potency estimates be coupled with information of chemical concentration in the thyroid tissue. In vivo thyroid levels of oxyfluorfen are modulated by chemical and physiochemical characteristics in addition to physiological processes of absorption, distribution, metabolism, and excretion (ADME). Inhibition potential and magnitude of THs thyroid synthesis can then be estimated in view of the in vivo thyroid tissue levels of oxyfluorofen in comparison to its estimated NIS inhibition potential in vitro using in vitro to in vivo extrapolation (IVIVE). IVIVE can be described as the process of calculating in vivo concentrations in target tissues (such as thyroid) that is equivalent to the in vitro dose used in high throughput assays where toxicological endpoints are observed (such as NIS inhibition), The purpose of this study is to develop an overall quantitative model incorporating oxyfluorfen ADME information and THs kinetics (synthesis, distribution, and catabolism). The overall model will be calibrated using in vitro data for NIS inhibition, and in vivo levels of the chemical and serum THs in rats. The calibrated model will then be extrapolated to estimated dose–response relationships for the inhibition of THs synthesis in humans.

## Materials and methods

### Integrative PBPK and THs kinetics modeling

**Fig. 2:**
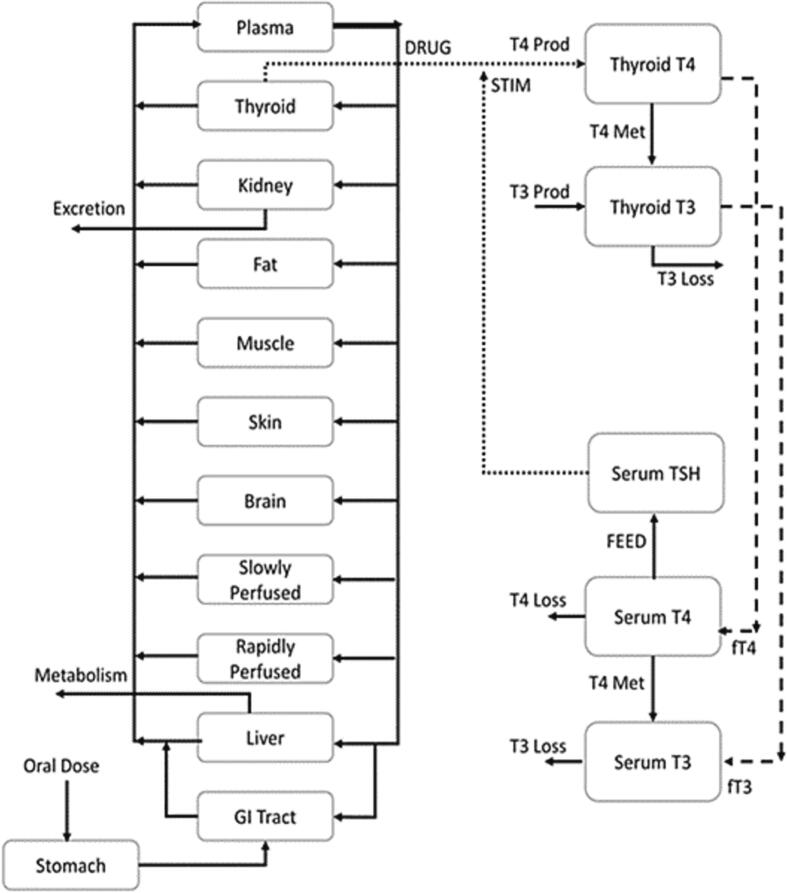
A Schematic of the overall computational model consisting of a physiologically based pharmacokinetic (PBPK) model for oxyfluorofen that is linked to thyroid hormones (THs) kinetics model in the thyroid and serum tissues. The chemical PBPK model is used to estimate levels of oxyfluorofen in thyroid gland. These tissue levels are then related to the synthesis of TH in the gland via a NIS inhibition mechanism using empirical equations derived from in vitro assays. Once formed in the thyroid, thyroxine (T4) and triiodothyronine are distributed to blood using their respective volumes of distributions. The synthesis of THs in thyroid tissue is stimulated (STIM) by serum levels of TSH which is regulated (FEED) by changes in serum levels of T4.

The overall rat and human modeling included a species-specific PBPK model integrated with a thyroid hormone kinetics one (Fig. 2). The PBPK model incorporated ADME physiological and biochemical processes that impact in vivo levels of Oxyfluorofen in the thyroid tissue in both species. The estimated in vivo thyroid tissue levels of Oxyfluorofen are then compared to experimentally derived in vitro quantitative relationship for inhibition potential to NIS by the chemical. In turn, the estimated in vivo inhibition potential is linked to THs kinetics model for the synthesis, distribution, metabolism, and excretion of THs for the prediction of serum hormone levels. Initially, the THs kinetics model was calibrated against experimental rat data. The overall calibrated rat model was then extrapolated to generate estimate T4 serum levels in humans exposed to daily doses of Oxyfluorfen.

### PBPK rat and human models

The PBPK model consisted of tissue compartments for: thyroid, GI tract, liver, muscle, slowly perfused, rapidly perfused, kidney, skin, and brain (Fig. 2). Oxyfluorfen oral dose was adjusted to account of increasing body weight in rats using experimental data. Once given orally, the chemical is then absorbed into the system from GI tract lumen described by using a first order constant k_a_ as follows:

$$\frac{dA_{\mathrm{stomach}}}{\mathrm{dt}}=-k_{a}*A_{\mathrm{stomach}}$$

Where $\frac{dA_{\mathrm{stomach}}}{\mathrm{dt}}$, and $A_{\mathrm{stomach}}$ are the rate and amount of chemical in gut lumen, respectively. The rate of chemical amount mass balance in the GI tissue is given by:

$$\frac{dA_{\mathrm{GI}}}{\mathrm{dt}}=Q_{\mathrm{portalvein}}*\left(C_{\mathrm{art}}-\frac{C_{\mathrm{GI}}}{pc_{\mathrm{GI}}}\right)+k_{a}*A_{\mathrm{stomach}}$$

Where $dA_{\mathrm{GI}}/dt$ is the mass balance rate of chemical in the GI tissue, $Q_{\mathrm{portalvein}}$ is the portal vein blood flow, $C_{\mathrm{art}}$ is the arterial blood concentration of the chemical, $C_{\mathrm{GI}}$ is the concentration of chemical in the GI tissue, and $pc_{\mathrm{GI}}$ is the tissue/blood partition coefficent of the chemical. The compartments representing muscle, slowly perfused, rapidly perfused, skin, fat and brain utilized a tissue specific blood flow and tissue/blood partition coefficient to predict the concentration of the chemical in each organ as follows:

$$\frac{dA_{x}}{\mathrm{dt}}=Q_{x}*\left(C_{\mathrm{art}}-\frac{C_{x}}{pc_{x}}\right)$$

Where $\frac{dA_{x}}{\mathrm{dt}}$ descride the rate of chemical amount in tissue x, $Q_{x}$ is the rate of blood flow to tissue x, $C_{\mathrm{art}}$ is the arterial chemical concentration, $C_{x}$ is the chemical concentration of tissue x, and $pc_{x}$ is the tissue: blood partition coefficient.

The mass balance equation describing the tissue concentration of Oxyfluorfen in the liver is given by the following equation:

$$\frac{{\mathrm{dA}}_{\mathrm{liver}}}{\mathrm{dt}}=Q_{\mathrm{artliver}}*C_{\mathrm{art}}-Q_{\mathrm{venliver}}*\frac{C_{\mathrm{liver}}}{pc_{\mathrm{liver}}}+Q_{\mathrm{portalvein}}*\frac{C_{\mathrm{GI}}}{pc_{\mathrm{GI}}}-CLR*\frac{A_{\mathrm{liver}}}{V_{\mathrm{liver}}*pc_{\mathrm{liver}}}$$

Where $\frac{{\mathrm{dA}}_{\mathrm{liver}}}{\mathrm{dt}}$ is the rate of chemical amount in the liver, $Q_{\mathrm{artliver}}$ is the blood flow to liver, $Q_{\mathrm{venliver}}$ is the venous blood leaving the liver is equal to sum of portal vein and arterial blood flow to the tissue, $C_{\mathrm{liver}}$ is the concentration of chemical in the liver tissue, $pc_{\mathrm{liver}}$ is the liver tissue/blood partition coefficient, $pc_{\mathrm{GI}}$ is the GI tissue/blood partition coefficent, CLR is the metabolic clearance rate of the chemical in the liver, $A_{\mathrm{liver}}$ is the amount of chemical in the liver tissue, and $V_{\mathrm{liver}}$ is the volume of liver tissue.

The kidney compartment included a term for excretion that is governed by the glomerular filtration rate (GFR) and the unbound fraction of chemical in the blood (frac).

$$\frac{{\mathrm{dA}}_{\mathrm{kidney}}}{\mathrm{dt}}=Q_{\mathrm{kidney}}*\left(C_{\mathrm{art}}-\frac{C_{\mathrm{kidney}}}{pc_{\mathrm{kidney}}}\right)-GFR*frac*C_{\mathrm{kidney}}$$

Where $\frac{{\mathrm{dA}}_{\mathrm{kidney}}}{\mathrm{dt}}$ is rate of chemical amount in the kidney tissue, $Q_{\mathrm{kidney}}$ is the blood flow to kidney, $C_{\mathrm{kidney}}$ is the concentration of chemical in the kidney tissue, $\mathrm{andp}c_{\mathrm{kidney}}$ is the kidney tissue/blood partition coefficent.

The thyroid model was split into two compartments: one for thyroid blood and the other for thyroid tissue where THs synthesis takes place.

Thyroid blood compartment is described as follows:

$$\frac{dA_{\mathrm{thyblood}}}{\mathrm{dt}}=Q_{\mathrm{thy}}*\left(C_{\mathrm{art}}-C_{\mathrm{thyblood}}\right)+PA_{\mathrm{thy}}*\left(\frac{C_{\mathrm{thytissue}}}{{\mathrm{pc}}_{\mathrm{thy}}}-C_{\mathrm{thyblood}}\right)$$

Thyroid tissue compartment is described as follows:

$$\frac{dA_{\mathrm{thytissue}}}{\mathrm{dt}}=-PA_{\mathrm{thy}}*\left(\frac{C_{\mathrm{thytissue}}}{{\mathrm{pc}}_{\mathrm{thy}}}-C_{\mathrm{thyblood}}\right)$$

Where $\frac{dA_{\mathrm{thyblood}}}{\mathrm{dt}}$, and $\frac{dA_{\mathrm{thytissue}}}{\mathrm{dt}}$ are the mass balance rate of amount of the chemical in thyroid blood and tissue, respectively. $Q_{\mathrm{thy}}$ is the blood flow to the thyroid tissue. $C_{\mathrm{thyblood}}$, and $C_{\mathrm{thytissue}}$ are the concentration of the chemical in thyroid blood, and tissue, respectively. The parameters ${\mathrm{pc}}_{\mathrm{thy}}$, and $PA_{\mathrm{thy}}$ are the thyroid tissue/blood partition coefficient, and the permeability area constant for diffusion, respectively.

### Ths kinetics model

THs hormone kinetics was mathematically described like earlier published models (Ekerot et al., 2013, Handa et al., 2021). Production of T4 in thyroid tissue was described by the following equation:

$$\frac{\mathrm{dAT}4_{\mathrm{thy}}}{\mathrm{dt}}=prod_{T4}*Inhib*stim-met_{T4thy}*AT4_{\mathrm{thy}}*fr-f_{T4srm}*AT4_{\mathrm{thy}}$$

And for T3 production in thyroid:

$$\frac{\mathrm{dAT}3_{\mathrm{thy}}}{\mathrm{dt}}=prod_{T3}*Inhib+met_{T4thy}*AT4_{\mathrm{thy}}*fr-met_{T3thy}*AT3_{\mathrm{thy}}-f_{T3srm}*AT3_{\mathrm{thy}}$$

Where $\frac{\mathrm{dAT}4_{\mathrm{thy}}}{\mathrm{dt}}$, and $\frac{\mathrm{dAT}3_{\mathrm{thy}}}{\mathrm{dt}}$ are the rate of change in T4 and T3 amounts in the thyroid tissue, respectively. The parameters $\mathrm{pro}d_{T4}$, and $\mathrm{pro}d_{T3}$ are the body weight scaled production rates of T4 and T3 in the thyroid, respectively. $\mathrm{me}t_{T4thy}$ is the first order rate constant for T4 conversion into T3 in the thyroid, while the rate of T3 metabolism in the thyroid is described by $\mathrm{me}t_{T3thy}$ rate constant. The constant fr is the fraction of T4 that converts to T3 which is set equal to 0.22 (Silva and Matthews, 1984). The rate at which T4 and T3 enters the blood is described using first order rate constants $f_{T4srm}$, and $f_{T3srm}$, respectively. The variable Inhib incorporates the inhibition of Oxyfluorfen on T4 productions while stim represents the inverse correlation between TSH and T4 as was given earlier by Ekerot et al. (2013).

The levels of T4 and TSH depend on feedback loops that regulate each other. Increased TSH levels stimulate thyroid T4 production via the stim term. Decreased serum plasma T4 levels stimulate TSH production through the variable feed1, and lowers the turnover rate of TSH through the term feed2. The interaction between T4 and TSH is given by the following equations:

$$\mathrm{stim}={{(C}_{\mathrm{TSH}}/TSH_{\mathrm{BL}})}^{NF3}$$

$$feed1={\frac{T4_{\mathrm{BL}}*VD_{T4}}{\mathrm{AT}4_{\mathrm{srm}}}}^{NF1}$$

$$feed2={\frac{\mathrm{AT}4_{\mathrm{srm}}}{T4_{\mathrm{BL}}*VD_{T4}}}^{NF2}$$

$$\frac{\mathrm{dATSH}}{\mathrm{dt}}=kin_{\mathrm{TSH}}*feed1-k_{\mathrm{TSH}}*feed2*ATSH$$

Where $T4_{\mathrm{BL}}/TSH_{\mathrm{BL}}$ represents the ratio of T4/TSH concentration, $VD_{T4}$ is the volume of distribution for T4, $\mathrm{ki}n_{\mathrm{TSH}}$ is the initial zero order production rate of TSH, and $k_{\mathrm{TSH}}$ is first order rate constant for loss of TSH. NF1, NF2, and NF3 are power constants adopted from Ekerot et al. (2013).

Thyroid hormones enter systemic circulation from thyroid tissue as given by the following equations:

$$\frac{\mathrm{dAT}4_{\mathrm{srm}}}{\mathrm{dt}}=f_{T4srm}*AT4_{\mathrm{thy}}-met_{T4srm}*AT4_{\mathrm{srm}}*fr-loss_{\mathrm{srm}}*AT4_{\mathrm{srm}}$$

$$\frac{\mathrm{dAT}3_{\mathrm{srm}}}{\mathrm{dt}}=f_{T3srm}*AT3_{\mathrm{thy}}+met_{T4srm}*AT4_{\mathrm{srm}}*fr-met_{T3srm}*AT3_{\mathrm{srm}}$$

Where $\frac{\mathrm{dAT}4_{\mathrm{srm}}}{\mathrm{dt}}$, and $\frac{\mathrm{dAT}3_{\mathrm{srm}}}{\mathrm{dt}}$ are the rate of change in T4 and T3 amounts in the serum, respectively. $\mathrm{AT}4_{\mathrm{srm}}$, and $\mathrm{AT}3_{\mathrm{srm}}$ are the amounts of T4 and T3 in serum, respectively. The parameters $\mathrm{me}t_{T4srm}$, and fr are the first order constant depicting the systemic transformation of T4 into T3, and the fraction of T4 that can be converted to T3, respectively. The parameters $\mathrm{los}s_{\mathrm{srm}}$ and $\mathrm{me}t_{T3srm}$ are first order constants describing systemic loss of T4 and T3, respectively.

### NIS inhibition by Oxyfluorfen

**Fig. 3:**
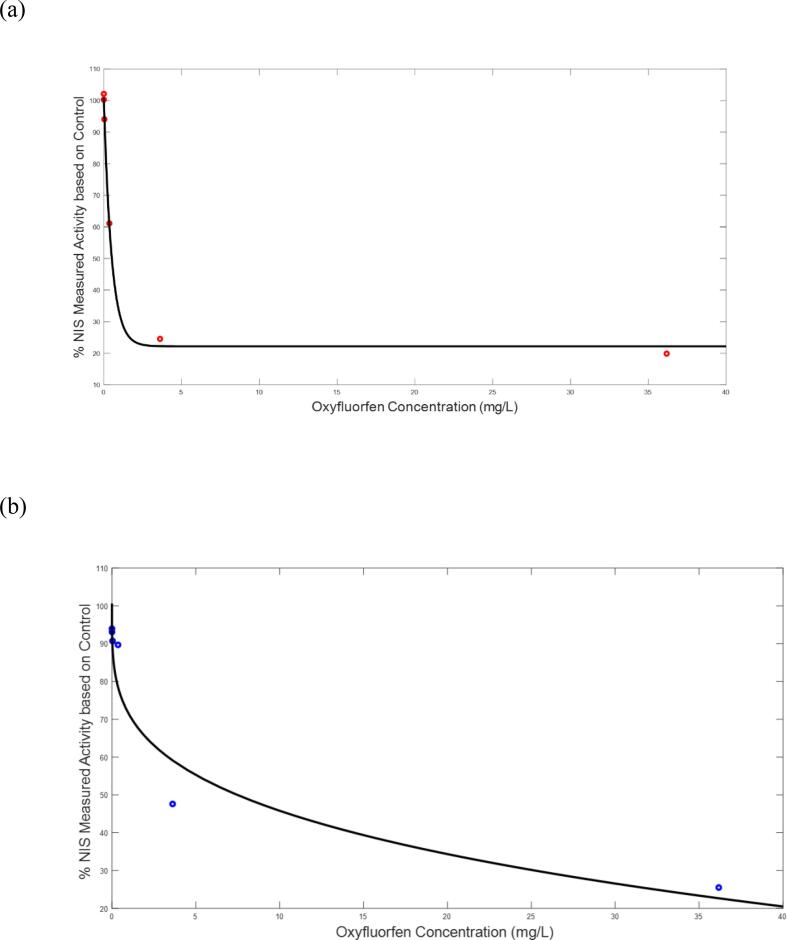
A graph of the empirical mathematical equations’ simulations against in vitro data for NIS inhibition. for (a) Rats, and (b) Humans cells. Data in both graphs are obtained from Buckalew et al. (2020). X-axis displays in vitro concentration of oxyfluorofen.

Inhibition of NIS by Oxyfluorfen in a dose–response manner was demonstrated in vitro in both human and rodent thyroidal cells (Buckalew et al., 2020). Information from the in vitro study was fitted to a mathematical function describing the dose–response relationship for rats and humans (Fig. 3.) The equations describing these in vitro relationships are given as follows where *CBthy* is the concentration of chemical in the thyroid cells (in vitro) or tissue (in vivo) as predicted from the Oxyfluorfen PBPK model.

## Inhib_rat_ = 0.788*exp(-2.006*CBthy) + 0.2218

Inhib_human_ = -0.2917*CBthy^.2739^ + 1.006.

### Integrative model parameters

The physiological parameters for both human and rat were obtained from Brown et al. (1997). These parameters are given in Table 1 and Table 2 for the human and rat, respectively. Tissue to blood partition coefficients for Oxyfluorfen were based on tissue content and physiochemical properties as calculated using GastroPlus 9.8 (SimulationsPlus®) software (Table 3). Oral absorption rate (Ka) for rats was fit to experimental data and assumed to be similar for humans. The hepatic clearance rate (CLR) for rats was fitted to data. The rodent parameter was then scaled to humans based on bodyweight. The glomerular filtration rate (GFR) for rats was obtained from Carrara et al. (2016), while the GFR value for humans were obtained from an average found in Delanaye et al. (2012). For rats, CLR and GFR values were adjusted based on bodyweight at termination of experiment. Table 4 is a listing of Oxyfluorfen biochemical parameters used in the PBPK models. Parameters for TH kinetics with their sources are listed in Table 5.

**Table 1:** Rat physiological parameter values1.

Parameter	Rat Value
Body Weight (kg)	Varies
Cardiac Output (L/hr/${\mathrm{kg}}^{.75}$)	0.235*BW^.75^*60
**Tissue Volumes (Fraction of Body Weight)**	
Plasma	0.074
Thyroid Total	0.00005
Thyroid Blood	0.18
Fat	0.070
Muscle	0.4043
Skin	0.1903
Liver	0.05
GI tract	0.026925
Kidney	0.0073
Brain	0.0057
Slowly Perfused Tissues	0.073^2^
Rapid Tissues	0.122^3^
**Blood Flow Rates (Fraction of Cardiac Output)**	
Thyroid	0.0027
Fat	0.070
Muscle	0.278
Skin	0.058
Liver	0.174
Portal Vein	0.153
Hepatic Artery	0.021
Kidney	0.141
Brain	0.020
Slowly Perfused Tissues	0.0823
Rapidly Perfused Tissues	0.3453

1 All parameters were obtained fromBrown et al. (1997).

2 Assumed to mainly consist of bone.

3 Calculated to account for remaining body weight.

**Table 2:** Human physiological parameter values1.

Parameter	Human Value
Age(years)Body Weight (kg)	30 80
Cardiac Output (L/hr/${\mathrm{kg}}^{.75}$)	−6.846*log10(age) + 16.775
**Tissue Volumes (Fraction of Body Weight)**	
Plasma	0.079
Thyroid Total	0.00005
Thyroid Blood	0.18
Fat	0.214
Muscle	0.40
Skin	0.037
Liver	0.026
GI tract	0.017
Kidney	0.004
Brain	0.02
Slowly Perfused Tissues	0.143^2^
Rapid Tissues	0.142^3^
**Blood Flow Rates (Fraction of Cardiac Output)**	
Thyroid	0.016
Fat	0.052
Muscle	0.191
Skin	0.058
Liver	0.227
Portal Vein	0.181
Hepatic Artery	0.046
Kidney	0.175
Brain	0.114
Slowly Perfused Tissues	0.042^2^
Rapidly Perfused Tissues	0.081^3^

1 All parameters obtained fromBrown et al. (1997).

2 Assumed to mainly consist of bone.

3 Calculated to account for remaining body weight.

**Table 3:** Blood-Tissue Partition Coefficients for Oxyfluorfen for Rat and Human PBPK model^1^.

Parameter	Rat Values	Human Values
Thyroid	8.5	8.5
Muscle	9.5	9.5
Skin	10.86	10.86
Fat	50.4	50.4
Liver	9.5	9.5
Kidney	9.5	9.5
Brain	20.31	20.31
Slowly Perfused	4.71	4.71
Rapidly Perfused	9.7	9.7
GI	9.5	9.5

1 Values were obtained from GastroPlus 9.8 (SimulationsPlus®).

**Table 4:** Oxyfluorfen Specific Biochemical Parameters in the Rat and Human.

Parameters	Units	Rat Value	Human Value
PAThy	L/hr	0.005^5^	0.005^6^
CLR	L/hr	1.275*(Avg.BW)^0.75^5^	118^1^
Ka	1/hr	0.00375^5^	0.00375^6^
frac	%	0.08^2^	0.08^2^
GFR	L/hr	60e-2*BW^3^	107^4^

1 Calcuated from in vitro hepatic clearance of 7.88 µl/min.10^6^ cells (Wetmore et al., 2012).

2 Obtained from GastroPlus 9.8 (SimulationsPlus®).

3 Obtained fromCarrara et al. (2016).

4 Obtained fromDelanaye et al. (2012).

5 Fit to rat data.

6 Assumed like the rat value.

**Table 5:** Thyroid Hormone Kinetic Model Parameters.

Parameters	Units	Rat Value	Human Value
**T4/T3 in Thyroid**			
fr	Unitless	0.22^8^	0.22^8^
T4prod	1/hr	3.68e-4*rat_BW^0.66^1^	0.0416^2^
T3prod	1/hr	3.43e-5*rat_BW^0.66^1^	2.7e-3^2^
T4met	1/hr	0.065^1^	0.065^1^
T3met	1/hr	0.045^1^	0.045^1^
**Basal levels**			
T4_BL	mg/L	208^3^	288^4^
T3_BL	mg/L	18^3^	33.4^4^
TSH_BL	mg/L	2.5e-3^3^	5e-3^4^
T4_BL_srm	mg/L	58.4e-3	85e-3^4^
T3_BL_srm	mg/L	1.26e-3	1.50e-3^4^
**TSH Parameters**			
k_TSH	1/hr	5e-6^3^	5e-6^3^
kin_TSH	mg/hr	TSH_BL * k_TSH	TSH_BL*k_TSH
NF1	Unitless	2.53^5^	2.53^5^
NF2	Unitless	1.9^5^	1.9^5^
NF3	Unitless	0.11^5^	0.11^5^
**T4/T3 in Serum**			
fT4	1/hr	0.13*rat_BW^0.66^1^	0.06^6^
fT3	1/hr	0.04*rat_BW^0.66^1^	0.02^6^
T4met_srm	1/hr	0.085^1^	0.085^1^
T3met_srm	1/hr	0.045^1^	0.045^1^
T4loss_srm	1/hr	0.045^1^	0.045^1^
VDT4	L/kgBW	0.149*Avg.BW^7^	0.149*Avg.BW^7^
VDT3	L/kgBW	1.62*Avg.BW^7^	1.62*Avg.BW^7^
VDTSH	L/kgBW	0.149*Avg.BW^7^	0.149*Avg.BW^7^

1 Fit to rat thyroid and serum data.

2 Human values obtained fromWiersinga et al. (2012).

3 Set to values obtained fromHanda et al. (2021).

4 Calculated fromKinjo et al. (2002).

5 Obtaiend fromEkerot et al. (2013).

6 Set similar to rat values obtained fromHanda et al. (2021).

7 Obtained fromDubois and Dussault (1977).

8 Obtained fromSilva and Matthews (1984).

## Experimental data

The PBPK models combined with the THs kinetics models were calibrated with experimental data provided via personal communication and illustrated in a co-submitted paper. In their experiment, adolescent Sprague Dawley rats were given Oxyfluorfen daily for 8 days. Rats were gavaged a daily single oxyfluorfen dose of 0.8125, 1.625, 3.25, 7.5, 15, 31.25, and 62.5 mg/kg in methyl cellulose (1 %) suspension. Levels of Oxyfluorfen were measured in thyroid tissue, and serum. THs levels for T4 and T3 were measured in serum at each administered dose.

### Model simulations and sensitivity analysis

All model simulations were conducted using MATLAB R2022A (MathWorks®). Basal Levels for rat THs and TSH in thyroid tissue and serum were obtained from experimental data and literature (Handa et al., 2021, Hassan et al., 2020). For Humans, basal levels for T4 and T3 in serum were obtained from Kinjo et al. (2002). Basal human thyroid tissue levels for THs were scaled using serum levels based on ratio of thyroid to serum TH levels in rats. For human serum TSH, basal levels were assumed similar to ones in rats given that literature TSH activity values for rats fall within the range given for humans (Hu et al., 2017, Solanki et al., 2013).Whenever applicable, parameters were estimated by fitting model simulations to data when predictions were within 2 fold or less different from experimental and control data using criteria as defined by World Health Organization (WHO) (WHO, 2010).

Using similar approach to our earlier publication (Leonard et al. 2016), formulation of sensitivity analysis for the both the PBPK and TH kinetics model equations was performed using the automatic differentiation package for MATLAB written by Martin Fink and Adam Attarian and available at MATLAB central (https://www.mathworks.com/matlabcentral/; Accessed February 19, 2016). The generated system of sensitivity equations was integrated with the model equations using MATLAB’s ODE solver. The sensitivity coefficients were normalized by dividing the value of the state variable, and multiplying by the parameter for which the derivative was being determined. Normalizing the sensitivity coefficients ensures that observed changes are equivalent regardless of the magnitude of the parameter or the state variable. Results of the sensitivity analysis for chemical thyroid concentration using the PBPK model show that parameters most sensitive to disposition of the chemical in the thyroid tissue were diffusion constant PA_thy_, oral absorption constant Ka, and blood/tissue partition coefficients for the liver and thyroid. For T4 serum levels, most sensitive parameters were related to systemic metabolism of the hormone, and its conversion to T3. For T3 serum levels, most sensitive parameters were related to T4 and T3 systemic metabolism in serum, and T4 conversion rate to T3.

## Results

### Oxyfluorfen levels in thyroid and blood

**Fig. 4:**
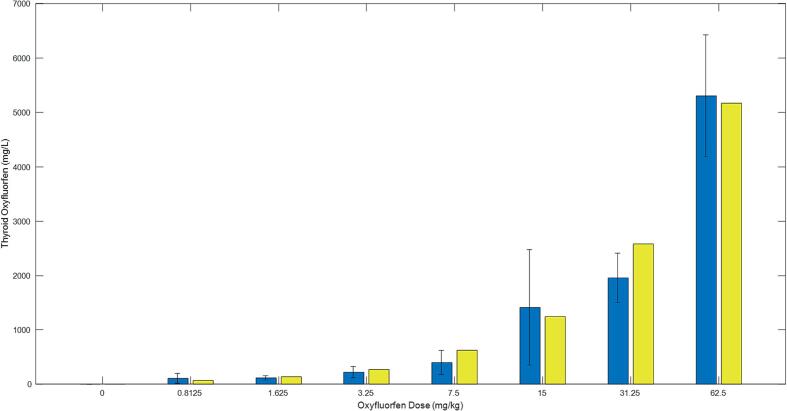
A graph for overall model simulations in comparison to data (bars with standard deviation) for the levels of oxyfluorfen in thyroid tissue in rats. Rats were administered the chemical in drinking water at different levels (x-axis) for 8 days. The data and model predictions were determined at the end of the 8 days. Data and experimental details were obtained via personal communication and is presented in a co-submitted manuscript (Stoker et al.).

**Fig. 5:**
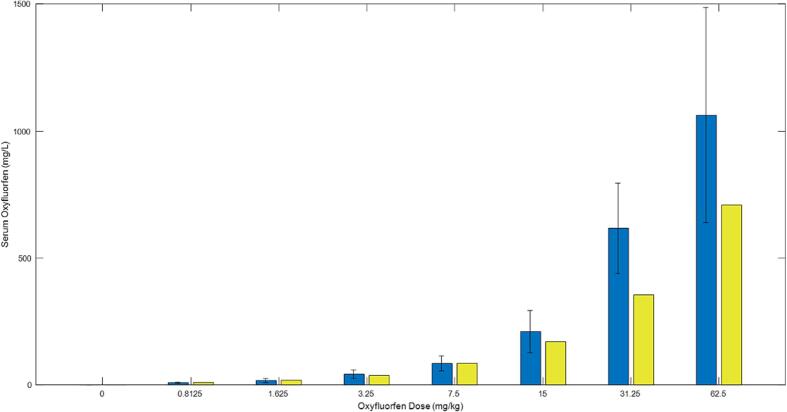
A graph for overall model simulations in comparison to data (bars with standard deviation) for the serum levels of oxyfluorfen in rats. Rats were administered the chemical in drinking water at different levels for 8 days. The data and model predictions were determined at the end of the 8 days. Data and experimental details were obtained via personal communication and is presented in a co-submitted manuscript (Stoker et al.).

Fig. 4, Fig. 5 display the model simulations and experimental data in thyroid tissue and plasma, respectively. As the administered doses of Oxyfluorfen increased, the levels of the chemical in both thyroid tissue and plasma also increased. The levels in thyroid tissue were almost three times higher than those in plasma. The model simulations followed the trend shown by the experimental data in both thyroid tissue and plasma.

### Ths hormones levels in blood

**Fig. 6:**
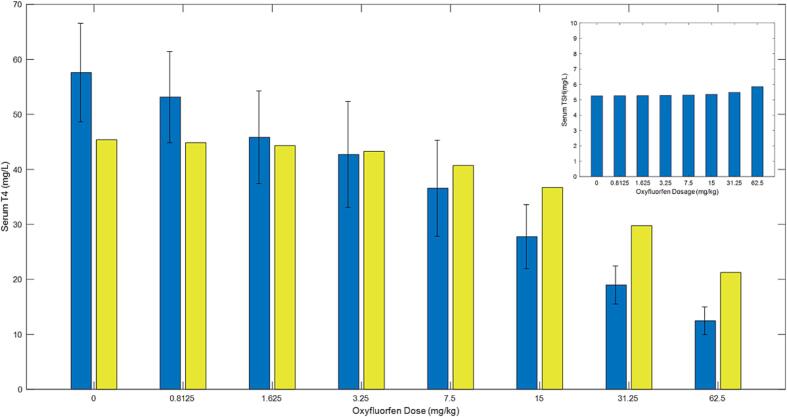
A graph for overall model simulations in comparison to data (bars with standard deviation) for serum T4 levels in rats given oxyfluorofen in drinking water. The insert graph shows model predicted levels of serum TSH for each given oxyfluorfen dose in the x-axis. Rats were administered the chemical in drinking water at different levels for 8 days. Data and model simulations were determined at the end of the 8 days. Data and experimental details were obtained via personal communication and is presented in a co-submitted manuscript (Stoker et al.).

**Fig. 7:**
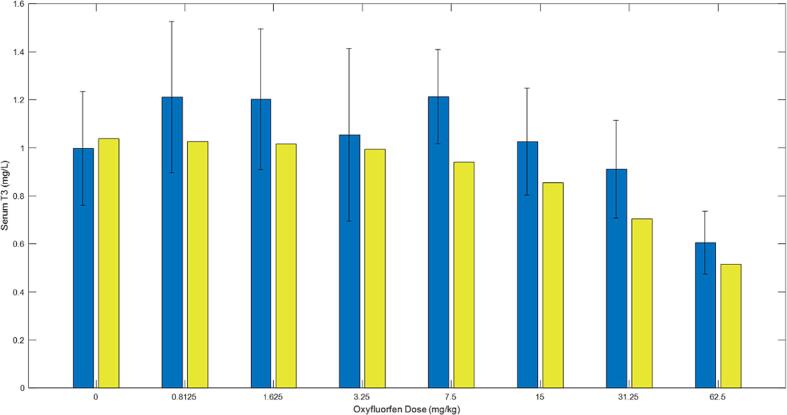
A graph for overall model simulations in comparison to data (bars with standard deviation) for serum T3 levels in rats given oxyfluorofen in drinking water. Rats were administered the chemical in drinking water at different levels (x-axis) for 8 days. Data and model simulations were determined at the end of the 8 days. Data and experimental details were obtained via personal communication and is presented in a co-submitted manuscript (Stoker et al.).

As the dose of Oxyfluorfen increased, the experimental T4 levels in the serum decreased. This trend was reflected in the model simulations, as demonstrated in Fig. 6. The experimental and simulated data for T3 are displayed in Fig. 7. Unlike T4, experimental T3 levels in the serum did not decrease across all doses, with drops only observed at higher dose levels (above 7.5 mg/kg). Although the dose–response behavior by the model simulations did not match with the experimental data at the lower dose, the overall predictions were within a 2-fold difference from the experimental data for T3 serum levels in rats exposed to Oxyfluorfen (Fig. 7) The results of the simulations and experimental data suggest that T4 is more susceptible to chemical stress, while T3, which is partially derived from T4, is affected only at higher doses.

### Predictions of TH serum levels for human Oxyfluorfen exposure

**Fig. 8:**
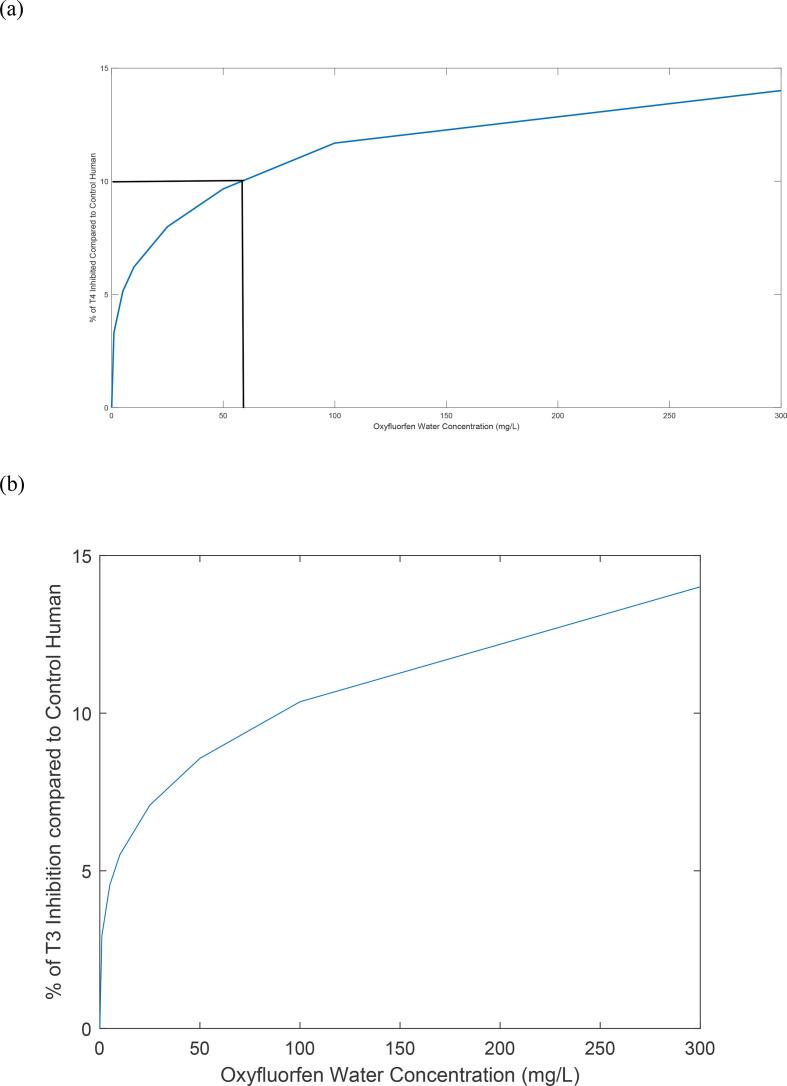
(a) A graph of the human overall model prediction of T4 serum levels precent inhibition at different oral daily doses of Oxyfluorfen in drinking water. A daily drinking water of oxyfluorfen at 57 mg/l is predicted to result in 10 % drop of T4 from background levels (b) A graph of the human overall model prediction of T3 serum levels precent inhibition at different oral daily doses of oxyfluorfen in drinking water. A daily drinking water of oxyfluorfen at 89 mg/l is predicted to result in 10 % drop of T3 from background levels.

The human overall model was utilized to forecast the decrease in T4 and T3 levels in response to long-term oral exposure to Oxyfluorfen in drinking water. The predicted decrease in T4, and T3 serum levels from their normal values in response to oral doses of Oxyfluorfen is displayed in Fig. 8. As shown in the figure, there is a correlation between the drop in T4 and T3 levels and the increase in the dose of Oxyfluorfen. This decrease slows down as the dose increases for predicted serum T4, and to a lesser extent for predicted serum T3, highlighting the interplay between pharmacokinetic and thyroid hormone kinetic modulators such as tissue saturation of oxyfluorfen levels, T4 to T3 deiodination, and TSH positive feedback regulation. Based on the predicted relationships, a 24-hour oral dose for oxyfluorfen water concentrations of 57 mg/L, and 89 mg/L is expected to result in a 10 % decrease in serum levels of T4, and T3, respectively.

## Discussion

The presence of endocrine disrupting chemicals (EDCs) in the environment continues to be a top public health concern not only for effects on the regulation of androgen and estrogen pathways, but also thyroid hormone homeostasis. Thyroid hormones regulate an array of physiological processes that are essential for metabolism, cardiovascular function, bone maintenance, as well as fetal and post-natal neurodevelopment. Regulatory agencies have traditionally relied on in vivo testing strategies using rodent models to identify thyroid disruption chemicals (TDCs) by their ability to suppress serum circulating levels of THs. However, the application of these strategies to human health risk assessment is limited due to the large number of untested chemicals in commerce, time, cost, and feasibility of obtaining in vivo testing data. These shortcomings precipitated the need for faster, cheaper high-throughput (HTP) in vitro screening approaches (Kavlock et al., 2012, Thomas et al., 2019).

HTP screening experiments can be strategically selected based on molecular initiating events (MIEs) along biochemical pathways related to disruptions of thyroid hormones hemostasis in serum (Tollefsen et al., 2014). An example for MIEs related to the synthesis of thyroid hormones, where screening HTP in vitro assay exist, are the inhibition of the thyroid peroxidase (TPO) or NIS symporter. Information obtained from these HTP assays are useful in screening chemicals for their potential to disrupt TH synthesis. However, application of the in vitro HTP data to chemical exposure and health risk assessment of TDCs requires their translation to in vivo estimates of THs levels in serum and target tissues such as a developing pre and postnatal brain. This necessitates the estimation of TDCs in vivo thyroid tissue levels where TH synthesis inhibition takes place. In turn, chemical in vivo thyroid levels are controlled by chemical pharmacokinetic determinants such as absorption, distribution, metabolism, and excretion (ADME) whereas THs in vivo serum levels are influenced by TH kinetics of synthesis, distribution, catabolism, and metabolism. An integrative computational model including chemical specific physiologically based pharmacokinetic (PBPK) and TH kinetics models provides a mechanistic quantitative approach to translate thyroidal in vitro HTP assays to in vivo measures of circulating THs serum levels. Several integrative quantitative models in literature exist where chemical specific ADME determinants are linked to hypothalamus-pituitary-thyroid (HPT) axis. Iodine and/or perchlorate PBPK models were used to quantitatively assess the impact of diet or chemical exposure on the inhibition of sodium/iodine symporter (NIS) and its impact on THs serum levels (Clewell et al., 2007, Fisher et al., 2013, Merrill et al., 2005). Another model that included NIS and TPO inhibition as a mechanism for chemical interaction with the HPT axis was developed by Willemin and Lumen (2017). They developed a computational model linking levels of thiocyanate in the thyroid to predict serum THs levels in rats and humans. We previously published a similar modeling approach integrating chemical PBPK to TH kinetics for the prediction of THs serum levels in vivo using HTP in vitro measures of THs synthesis inhibition by two well-known TPO inhibitors Propylthiouracil (PTU) and Methimazole (MMI) (Handa et al., 2021). Following similar approach to our earlier integrative modeling approach for TPO inhibition, we utilized HTP in vitro data for the NIS inhibition by the chemical Oxyfluorofen to predict THs serum levels in vivo in rats and humans.

Experimental results from the study on Oxyfluorfen levels in thyroid and blood suggest that increasing doses of Oxyfluorfen can lead to an increase in the levels of the chemical in both thyroid tissue and plasma. In particular, the levels in thyroid tissue were almost three times higher than those in plasma. These findings are supported by both the experimental data and the integrative model simulations. Additionally, the results showed that increasing doses of Oxyfluorfen can lead to a decrease in experimental T4 levels in the serum. This trend was reflected in the model simulations. Unlike T4, experimental T3 levels in the serum did not decrease across all doses, with drops only observed at higher dose levels whereas model simulations showed a more robust dose–response. The resistance for T3 to significantly drop at the lower doses may be an outcome of the hormone being a product of T4, hence its depletion is secondary to T4, and may only be significant when a large drop of T4 is taking place at the higher doses. Our data and modeling simulations suggest that serum T4 levels are more sensitive indicators of exposure to NIS inhibitors than T3 levels, even though the latter is the bioactive hormone.

The IVIVE approach and computational modeling adopted here were based on in vitro high throughput assays for NIS inhibition, limited in vivo experimental data in rats, and species extrapolation to humans using allometric parameter scaling whenever applicable. Most of the pharmacokinetic parameters used in the overall model for rats and humans were either obtained or calculated from literature and relevant commercial modeling software, while the availability of in vivo experimental data in rats was used to calibrate the model for some of the thyroid kinetics model parameters. In humans, some of the parameters were obtained from the ones used in rats using allometric scaling methods in lieu of absence of human in vivo calibration data for the chemical. For the rat model, more detailed time, and dose–response experimental data for thyroid hormone levels in thyroid tissue and serum are needed to further evaluate the overall model predictions. For humans, there is a need to evaluate the model predictions against any in vivo data of oxyfluorfen serum levels and any resulting drops of serum thyroid hormones levels. Hence, application of the presented overall model to conduct cross-species extrapolation and estimate points of departures based on disruption of thyroid hormones serum levels in humans by oxyfluorofen exposure should be considered with caution**.** Nonetheless, the overall modeling approach provides a biologically based computational framework to conduct IVIVE based on in vitro high throughput data and can be further used to illustrate species related parameters and processes that may impact the estimation of such points of departures based on similar modes of action. Experimental concentrations of oxyfluorofen that result in a 50 % drop of NIS activity (IC50) in vitro were calculated from published data by Buckalew et al. (2020) as 0.8 and 0.7 mg/l for rats and humans, respectively. However, this in vitro closeness of the chemical potential for NIS inhibition in both species is not reflected by the magnitude of T4 in vivo serum drops as predicted by the overall computational model. When the overall model was used to identify an experimental oxyfluorfen applied dose in rats that will result in a 10 % drop of serum T4, the simulations resulted in an estimated daily dose of 7.5 mg/kg in drinking water. Assuming a human water consumption rate of 2L/day and 80 kg bodyweight, the equivalent daily dose to the 7.5 mg/kg in humans is 300 mg/L of daily oxyfluorofen exposure in drinking water. In contrast, the overall model predicted dose in water of oxyfluorofen that is needed to reach a 10 % drop of serum T4 in humans is predicted as 57 mg/l. Therefore, the IVIVE overall model predicted dose in humans is 5-fold lower than the one calculated based on rat data. This analysis highlights the important role of considering biological process such as pharmacokinetics and thyroid kinetics to conduct IVIVE for the purposes of estimating points of departure for adverse health outcomes based on in vivo drop of T4 levels in serum.

Disclaimer

This document was reviewed by the Center for Computational Toxicology & Exposure, Office of Research and Development, U.S. Environmental Protection Agency, and approved for publication. Approval does not signify that the contents reflect the views of the Agency, nor does mention of trade names or commercial products constitute endorsement or recommendation for use.

## CRediT authorship contribution statement

**Rhylee Decrane:** Software, Formal analysis, Data curation, Writing – review & editing. **Tammy Stoker:** Methodology, Visualization, Investigation, Data curation, Writing – review & editing. **Ashley Murr:** Data curation. **Jermaine Ford:** Data curation. **Hisham El-Masri:** Supervision, Methodology, Software, Writing – review & editing, Writing – original draft.

## Declaration of Competing Interest

The authors declare that they have no known competing financial interests or personal relationships that could have appeared to influence the work reported in this paper.

## Data Availability

Data will be made available on request.
